# Socio-Demographic and Leisure Activity Determinants of Physical Activity of Working Warsaw Residents Aged 60 to 69 Years

**DOI:** 10.2478/v10078-011-0085-y

**Published:** 2011-12-25

**Authors:** Elżbieta Biernat, Paweł Tomaszewski

**Affiliations:** 1 Warsaw School of Economics, Warsaw, Poland; 2 University of Physical Education, Warsaw, Poland

**Keywords:** physical activity, IPAQ, BMI, environmental factors, leisure activity, elderly

## Abstract

The purpose of the study was to assess factors determining physical activity in persons at the age of 60–69 years in an urban area. The study included 262 working residents of Warsaw at the initial period of old age. The study utilized a questionnaire consisting of two parts. The first part concerned recreational and touristic activities in the previous year. The second is a Polish version of IPAQ, assessing the respondents’ level of activity throughout the past week. Based on IPAQ results, the respondents were divided into physically active and inactive ones. The active group included people meeting moderate to vigorous physical activity, whereas the inactive group included people who took up no physical activity at all or those with a low physical activity level. The relations between taking up physical activity and the variables characterizing the demographic structure as well as touristic and recreational activity of the respondents were assessed with the use of a log-linear analysis. Out of the variables taken into account, age, education and participation in physical recreation proved to be significant factors in taking up activity by the elderly. The odds ratios computed for the analyzed variables indicate that the risk of being inactive increases over two times after exceeding 65 years of age; a risk of similar magnitude was also observed in case of less educated populations. Regular participation in physical recreation provides a four-times increase in the chances to achieve levels of physical activity sufficient to remain healthy.

## Introduction

Population of people over 60 has increased significantly over the last few years in Poland. According to the general population census of 2002, people over 60 contribute to about 15% of the general Polish population (Central Statistical Office, 2009b). It is estimated that in 2050, the percentage of seniors can reach over 35% of the population (Tobiasz-Adamczyk et al., 2009). This changing age structure of Poles forces actions that will give the elderly a chance for the so-called *successful aging* (good health at old age, satisfaction with life, self-acceptance, ability to adapt to modern environment; Lewis, 2005). A significant role is played here by functional fitness and self-dependence, as well as the ability to do basic (P-ADL - *activity of daily living*) and more complex, instrumental (I-ADL) day-to-day activities (Fries, 1998; Frymoyer, 1988). Functional fitness is influenced by many factors such as: medical history, medications in use, nutrition, obesity and the coexistence of metabolic disorders, but above all, physical activity.

Determining the correlates of participation in physical activity is a first step that may help to target at-risk populations and guide the development of preventive programs which is of utmost importance, especially in older adults. Among many factors related to seniors’ overall physical activity levels, associations with sociodemographic variables - such as gender, education and income levels were of interest to many authors (Sherwood and Jeffery, 2000; Trost et al., 2002; Wilcox et al., 2003). It was shown in general that education, marital status and social support were positively, while age negatively associated with participation in physical activity. On the other hand, strong cultural conditionings most fully manifest in advanced age result in becoming sedentary, in overestimating passive leisure, what in turn negatively affects participation in sport, tourism and recreation.

Therefore, apart from commonly applied methods of assessment in gerontological prevention (Katz et al., 1963; Lawton and Brody, 1988), measurements and monitoring of physical activity are more and more frequently applied. In smaller groups, it is possible to apply direct methods (e.g. calometric methods) for this purpose, whereas in larger populations - various types of questionnaires (Biernat and Stupnicki, 2005). These are helpful in e.g. assessing work carried out in one’s spare time, housework, activity related to professional work and recreation. Among these tools, the International Physical Activity Questionnaire (IPAQ) can be distinguished - especially recommended by international research societies i.e. European Health Interview Society (EUROHIS), European Physical Activity Surveyance System (EUPASS) and the European Social Survey 2002 (www.ess.nsd.uib.no) – unfortunately, still largely unknown and unused in Poland. The presented study applied the Polish version of IPAQ developed (and officially approved by the IPAQ Committee) by the author of this paper and by Stupnicki in 2004. Since this tool’s accuracy and reliability has been verified for people between 15 and 69 years old, this study only includes subjects at the age 60–69 - which, according to WHO criteria, is the initial period of old age.

Increased interest in issues related with successful aging is observed throughout the past few years, which results in the emergence of more and more studies, mainly covering functional fitness assessments and the quality of life of the elderly. The few studies that address the issue of physical activity of elderly people, largely concern the assessment of its levels, without however describing its determinants. Thus, the goal of this study was to assess the factors determining taking up physical activity by people at the age of 60–69. The results of this study may contribute to fill the gap presently observed in this field.

## Material and Methods

The study included 262 working Warsaw residents at the age of 60–69. The majority of them were people, whose professions offered the highest positions in international scales of prestige and economic status. Survey data was gathered with the use of direct, standardized interviews. The interviews were carried out by trained and supervised pollsters, according to a specific plan (the number of questions asked as well as the way of asking them were identical for all respondents). Randomization procedure consisted of two stages: in the first stage 3–10 institutions employing persons of specified profession were randomly selected out of all Warsaw’s institution of a given sector. In the second stage, about 20 employees from each of previously selected institution were randomly qualified for the study.

During the interview, data was gathered regarding sex, age, education, body height and body mass, as well as material and marital status of the respondents, which are possible determinants of physical activity. Adapting the WHO 5-years-interval classification of the elderly age, the respondents were divided into younger subjects (60–64 years) and older ones (65–69 years). Based on their body weight and height, their BMI was calculated, which served in classification of respondents into standard body mass/nutrition categories (normal, underweight, overweight, obese). Due to the domination of people with higher education among the respondents, other education categories were combined and compared to respondents with higher education. Based on Central Statistical Office data (http://www.stat.gov.pl/gus/5840_1630_PLK_HTML.htm), people who earned less or more than the average monthly earnings (about 2700 PLN; approx. 700 Euro) in the years when the survey was being carried out (2006–2009) were distinguished among the respondents. The respondents were also divided into those living in a relationship (marriage, concubinage) and those not living in partner relationships (widows/widowers, divorcees, singles). All variables which respondents were asked about have been considered in the analyses.

The characteristics of the tested subjects within particular categories are presented in Table 1. The questionnaire concerning physical activity consisted of two parts. The first part concerned recreational and touristic activity realized throughout the last year. This part of questionnaire was designed for the purpose of a larger research project (about 7000 Warsaw residents assessed) concerning conditionings of physical activity of an urban population. Accuracy and reliability of the questionnaire was verified in pilot studies conducted on a random sample of 300 subjects. It helped to gather information on the degree of participation and frequency of taking up various forms of recreation and tourism in one’s spare time. The part of the questionnaire pertaining to recreation consisted of two single-choice questions (concerning declared participation and mode of participation in recreation) and one free-answer (open) question on declared forms of recreation. The part devoted to touristic activities consisted of six closed questions (pertaining to participation in short- or long-lasting local or abroad trips and their objectives) and one open question specifying abroad destinations. In addition, due to the popularity of this form of recreation in this particular age group, the character of garden work (regular, irregular) was also established.

Based on the gathered data, the level of respondents’ participation in tourism was established (low, moderate and high), as well as the character of their participation (regular, irregular). Level of tourism participation was determined with the use of principal component analysis (PCA). Information on declared frequency of touristic activities (short, long and abroad trips) was reduced to one variable and subsequently categorized on the basis of respective quantiles. Finally, calculated variable – level of tourism participation – allowed classification of respondents into one out of 3 categories – low, moderate or high.

Participation in activities for at least 5 months, at least once weekly, was defined as regular; for several or more consecutive days, over 10 times in a season, as seasonal; several or more times a year, as occasional.

The second part of the questionnaire is a short version of IPAQ, which helped to gather information on the frequency and duration of all physical activity (intensive, moderate and walking) - undertaken by respondents throughout the last week. Based on those - and after standard calculations (Biernat et al., 2007; www.ipaq.ki.se) -the level of physical activity of the respondents was assessed as follows:.

- Low
  - no activity is reported; or
  - some activity is reported but not enough to meet moderate or high category
- Moderate
  - 3 or more days of vigorous activity for at least 20 min per day; or
  - 5 days or more of moderate intensity activity or waking for at least 30 min per day or
  - 5 or more days of any combination of waking, moderate or vigorous intensity activities achieving a minimum of 600 MET min-week^−1^
- High
  - 3 or more days of vigorous activities accumulating at least 1500 MET min-week^−1^; or
  - 7 or more days of any combination of waking, moderate or vigorous activities achieving a minimum of 3000 MET min-week^−1^.

Taking into account the WHO recommendations concerning the dose of physical activity necessary to maintain health, the respondents were divided into physically active (n = 177) and inactive (n = 85). The active group included people with moderate to high level of physical activity; the inactive group included people who took up no physical activity at all or had low levels of it.

According to the rules established by the IPAQ Committee (www.ipaq.ki.se), the study was only performed in March and November (2006–2009). The study excluded periods related to holidays. The study also excluded respondents who were ill during the past 7 days, were hospitalized, took rehabilitation activities, were on a sick leave, etc. The percentage of refusals to grant an interview was low and closed in the range of 3–5%.

The relations between taking up physical activity and variables characterizing the demographic structure and touristic and recreational activity of the respondents were assessed with the use of log-linear analysis. All variables which respondents were asked about have been considered in analyses. Due to methodological restrictions of log-linear modeling, as well as low cardinalities resulting from multidimensional combinations of variables, the analysis was performed separately for both factor groups; the first model (Model 1) included sex, body mass (based on BMI), age and education of the respondents, and the second one (Model 2) their material status, marital status and variables associated with touristic and recreational activity, as well as work in the garden. Analysis of simultaneous interaction tests for all studied variables (in both models) were performed first. In the second stage, based on partial and marginal correlations the significant correlates of physical activity were specified. Partial association tests the significance of deleting a particular effect from a model when all the remaining effects of the same order are already included. A significant chi-square value for an effect implies that the effect should be considered in models subjected for further analysis, deleting that effects reduces the adequacy of fit. Marginal association tests of the significance of deleting an effect from a model which contains only that particular effect (none of remaining effects of the same order are included). A significant result again implies that the given effect makes a difference in the adequacy of fit and should be considered in models to further evaluations. The final models were selected based on Akaike (AIC) criterion. The significance of associations between factors included in the analysis was assessed with the use of χ^2^ test. The results were presented with fractions calculated based on observed cardinality and odds ratios with 95% confidence intervals. The analyses were performed with the use of STATISTICA 8.0 PL statistics pack. While assessing the results, the level of p<0.05 was considered significant.

## Results

Descriptive statistics of physical activity variables recorded in active and inactive subjects are presented in Table 2. In the active group, practicing vigorous activities were declared by 36% of respondents, moderate activities by 77% and walking by nearly all of the studied subjects. In the inactive group the respective percentages were 8, 42 and 62%,. Additionally, active subjects prevailed in number of days and time spent weekly on practicing particular activities, but spent less time daily on sitting, the respective time being 416 and 523 min/day. It resulted in weekly energy expenditure which averaged 2208 MET min-week^−1^ in the active group, the amount being nearly seven times higher than the total activity observed in inactive subjects (323 MET min-week^−1^).

As a result of the performed analysis of simultaneous interaction tests for selected socio-demographic variables and physical activity, a satisfactory goodness-of-fit of the tested model was observed, taking into account two-way interactions (χ^2^ = 37,8; p = 0.004). Including three-way interactions had no significant impact on improving the model’s fitting (χ^2^ = 14,6; p = 0.88). Likewise, in case of the tested model of variables associated with leisure activity as well as marital and material status, the significant model included two-way interactions (χ^2^ = 54,5; p = 0,000), whereas including three-way interactions into the tested model did not improve its goodness-of-fit (χ^2^ = 24,9; p = 0,73). In case of analyzed models including two-way interactions, a satisfactory fitting with observed data was achieved: the probability values for maximum likelihood χ^2^ test significantly exceeding the level of 0,05 (p = 0,89 and p = 0,96 for model 1 and model 2 respectively).

As a result of analyses of partial and marginal chi-square associations made for both models, it was found that out of the variables in question, only age (χ^2^_partial_ = 5,70; p = 0,017), education (χ^2^_partial_ = 8,28; p = 0,004) and participation in physical activity (χ^2^_partial_ = 12,6; p = 0,001) proved to be significant factors determining taking up activity by elderly people. The odds ratios computed for the analyzed variables indicate that the risk of becoming inactive increases over two times after exceeding 65 years of age (Table 3).

A risk of comparable magnitude was also observed in case of less educated subjects. On the other hand, regular participation in physical recreation provides a four-times increase in the chances to achieve levels of physical activity sufficient to remain healthy. In reference to sex, various BMI categories, material status and work in the garden, the number of active and inactive people did not vary significantly, and in case of living in a relationship and the degree of participation in tourism, the percentages observed were almost identical (differences did not exceed 2%).

## Discussion

One of the major issues of industrialized countries is maintaining health and the quality of life of the elderly, thus maintaining their independence at an appropriate level. The level of physical activity - as one of the elements of functional fitness - plays a significant role here. It allows for the prevention and modification of many negative factors affecting the process of aging (King et al., 2000; Rejeski et al., 2002).

The results of the presented research show that about two thirds of Warsaw residents between 60–69 years (67%) are characterized by moderate to high level of physical activity, thus meeting the recommendations of American and European health organizations (Pate et al., 1995). A summary of these results with studies carried out among citizens over 55 years in 17 countries of the EU, belonging to various socio-professional groups and evaluating their health as good or very good show that 54% of the tested EU citizens are characterized by moderate, and 60% by a high degree of physical activity (Abu-Omar et al., 2004). Ruuskanen and Ruoppila (1995) observed that Finns between 65–69 years who rated their health as good, exercised more regularly and intensively (35%) than those who rated it as average or poor (17%). Despite their current health status and regardless of their position and education, the percentage of active people is smaller than in Warsaw population in all countries compared.

As it was shown, taking up physical activity decreases gradually with age (Schoenborn and Barnes, 2002). According to Krems et al. (2004), elderly people show reluctance towards taking any kind of physical effort. The presented studies show that 78% of physically active people at 60–64 years falls drastically to 22% at 65–69 years, causing a twofold increase in the risk of becoming inactive. Therefore, we can assume that the age of 65 is the limit where people go defensive on their health. The results are consistent with Ruuskanen’s and Ruoppil’s (1995) reports, which showed the percentage of men and women between 65–69 years taking up activity beyond daily life activities was 27% and 20% respectively. This percentage was reduced with the passing of years by 2–5% per 5 years, falling to the value of 5% of women at 80–84 years. The above observations suggest that promoting active lifestyle should already be stressed at the pre-retirement age - especially in societies, where restricted fitness or a low level of physical activity is observed.

In case of highly educated people, with prestigious professions or of a high social status - the phenomenon of decreasing percentage of active people with age is not as clearly observed as in other social groups (Jones et al., 1998). These people are more likely to follow the principles of a healthy lifestyle, care about their health and monitor their health status, take up sports and other types of activities, have wider knowledge on the hazards for their physical and mental wellness and are able to avoid these negative conditions and minimize their negative effects (Hawkins et al., 2004). This was confirmed in this study, where the results indicate that people with higher education were active over 5 times more frequently than people with lower education (85 vs. 15%). Such a large disproportion is the result of a significant over-representation of people with higher education, which is reflected in the high percentage of highly educated people in the inactive group as well (69 vs. 31%). However, the ratio between active and inactive people is significantly (about 2.5 times) larger among people with higher education than among other respondents, where this ratio is 1:1. Other analyzed socio-demographic factors, such as sex, marital status, income, or participation in tourism, had no significant impact on taking up physical activity. It is striking that the ratio between active and inactive people was also comparable in people with improper BMI, which characterized over 60% of the respondents. These observations are not in line with results of Kyle et al. (2004) who found that physically active subjects were less likely to have a high or very high body fat mass index (BFMI), and more likely to have low BFMI. In the study performed on 56–73 year old women (Gaba et al., 2009), the positive effect of physical activity was mostly pronounced in the changes of body fat content - the absolute and relative body fat proportion and the BFMI was higher in the inactive women than in the active ones and its proportion decreased in correlation with the intensity of physical activity (r = − 0.40; p < .05) and the number of steps per day (r = −0.50; p < .05). As the study covered quite a large sample of over 250 randomly selected Warsaw residents aged 60–69 years, the results may be considered representative and thus generalised. However, a certain limitation of the study, potentially distorting the image of physical activity correlates in elderly people, might have been low cardinalities in some categories of analysed variables (e.g. BMI).

Monitoring of anticipated results of the National Health Program (NHP) shows that extending the life expectancy of Polish women to 79 years and men to 70 years was largely due to the change in lifestyle, including increased physical activity taken up under recreational activities (www.pzh.gov.pl). However, Central Statistical Office (2009a) reports that despite NHP’s recommendations (increasing physical activity in elderly people up to 30% of their population) - only 20% does any kind of physical recreation. This is not the case with respondents from 15 EU countries and Americans: almost 65% of Europeans and almost 50% of Americans at 65–74 take up some kind of recreation (Martinez-Gonzales et al., 2001; Schoenborn and Barnes, 2002). The results of the presented studies are closer to the mentioned foreign reports and show that, among the active Warsaw residents (with a high or moderate degree of activity) taking up regular recreation was declared by 42%, whereas among the inactive group (with a low degree of activity) - only 14% of the respondents, which means that people who regularly participate in recreation have 4 times (OR 4.37) larger chances to qualify to moderate degree of activity and thus meeting WHO recommendations regarding dose of physical activity necessary to maintain health.

To conclude, the used log-linear modeling method allowed observing a significant association of age, education and recreational activity with taking up physical activity by people between 60–69 years. Moreover, lack of significant three-way (or higher) interactions implies that the interaction between the analyzed variables is the same for both categories of physical activity. As lack of exercise and overestimating passive leisure both have a strong cultural background and manifest most fully after 65 years with reducing activity and increasing dependence on others, it is crucial to maximize support programs for this social group as well as taking action not only by national institutions, but also various informal groups, non-governmental organizations as well as institutions of local authorities.

As this study suggests, physical capacity of elderly can be maintained through regular exercise and active tourism. It is worth remembering that civilization changes that are observed today in the Polish society will cause that in a dozen years or so, we will be dealing with an entirely different kind of seniors - well-educated, active, aware of their rights, wishing to fulfill their passions and interests in their old age as well. Therefore, we must already consider, diagnose and popularize solutions that will address the changing needs of this social group. Although the presented results put some light on the physical activity determinants of Warsaw’s older population, it seems reasonable to extend the scope of the future studies on other urban/rural populations and on other groups of seniors. Moreover, applying the long-version of IPAQ questionnaire would contribute to better understanding of recreational correlates of physical activity, although using this tool was shown to be difficult and not entirely reliable under Polish conditions (Biernat et al., 2007).

## Figures and Tables

**Table 1:** C*haracteristics of the subjects studied (n = 262)*

Variable	n	%
Sex		
Male	131	50%
Female	131	50%
Age		
60–64 years	192	73%
65–69 years	70	27%
BMI		
Normal	94	36%
Underweight	7	3%
Overweight	129	49,5%
Obese	30	11,5%
Higher education		
No	52	20%
Yes	209	80%
National average income (approx. 700 Euro)		
Below	161	69%
Above	74	31%
Living in a relationship		
No	64	25%
Yes	197	75%

Due to possible data deficiencies, the number of respondents may vary between individual variables

**Table 2:** Descriptive statistics of physical activity variables recorded in active and inactive Warsaw residents aged 60 – 69 years (percentages, mean ± SD and medians)

Variable	Active n = 177	Inactive n = 85
Vigorous activity	36%	8%
Number of days	2.6±1.8 (2)	1.4±0.5 (1)
Time spent weekly [min]	167.5±211.5 (90)	101.4±95.5 (60)
Moderate activity	77%	42%
Number of days	3.5±2.1 (3)	2.0±1.4 (2)
Time spent weekly [min]	267.9±414.4 (150)	57.9±37.9 (55)
Walking	94%	62%
Number of days	5.9±1.7 (7)	3.3±1.9 (3)
Time spent weekly [min]	288.9±338.2 (210)	76.7±44.5 (70)
Sitting [min/day]	415.6±175.1 (420)	523.1±175.4 (540)
MET Total [MET min-week^−1^]	2208±2582 (1422)	323±360 (325)

**Table 3:** Factors determining physical activity of people at the age 60–69 years (n=262) and odds ratios (OR) as well as 95% confidence intervals (95% CI) established for being active

Variable	Active		Inactive		p	OR	95% CI

	n	%	n	%			
Sex					0,235		
Male	84	47%	47	55%		1	-
Female	93	53%	38	45%		1,37	0,81 – 2,30
**Age**					**0,015**		
60–64 years	138	78%	54	64%		1	-
65–69 years	39	22%	31	36%		0,49	0,28 – 0,87
BMI					0,364		
Normal	58	33%	36	42%		1	-
Underweight	6	3,5%	1	1%		3,72	0,43 – 32,2
Overweight	91	52%	38	45%		1,49	0,85 – 2,61
Obese	20	11,5%	10	12%		1,24	0,52 – 2,95
**Higher education**					**0,003**		
No	26	15%	26	31%		1	-
Yes	150	85%	59	69%		2,54	1,37 – 4,73
National average income (approx. 700 Euro)					0,376		
Below	106	67%	55	72%		1	-
Above	53	33%	21	28%		1,31	0,72 – 2,39
Living in a relationship					0,795		
No	44	25%	20	24%		1	-
Yes	132	75%	65	76%		0,92	0,50 – 1,69
Level of participation in tourism					0,942		
Low	74	42%	35	41%		1	-
Moderate	23	13%	10	12%		1,09	0,47 – 2,53
High	80	45%	40	47%		0,95	0,54 – 1,64
**Participation in recreation**					**0,001**		
Irregular	103	58%	73	86%		1	-
Regular	74	42%	12	14%		4,37	2,21 – 8,62
Working in the garden					0,150		
Irregular	109	62%	60	71%		1	-
Regular	68	38%	25	29%		1,50	0,86 – 2,61

Significantly contributing factors are bolded. Due to possible data deficiencies, the number of respondents may vary between individual variables
